# Laboratory Bill for Study of Criminal, Pauper and Defective Classes

**Published:** 1903-04

**Authors:** 


### Laboratory Bill for Study of Criminal, Pauper and Defective Classes.

That there shall be established in the Department of Justice a laboratory for the study of the abnormal classes, and the work shall include, not only laboratory investigation, but also the collection of sociological and pathological data, especially such as may be found in institutions for the criminal, pauper, and defective classes, and generally in hospitals and schools. Said laboratory and work shall be in charge of a director, who shall be appointed by the President, and shall receive a salary of three thousand dollars per annum. He shall make a report once a year, directed to the Attorney General, which, with the approval of that officer, shall be published. For the proper equipment of said laboratory, and the rental, if necessary, of a suitable room therefor, there is hereby appropriated out of any money in the treasury, not otherwise appropriated, the sum of five thousand dollars, or so much thereof as may be required. And as so amended that the bill pass.

